# Chlorogenic Acid Ameliorates Damage Induced by Fluorene-9-Bisphenol in Porcine Sertoli Cells

**DOI:** 10.3389/fphar.2021.678772

**Published:** 2021-06-09

**Authors:** Shaoxuan Zhang, Boxing Sun, Dali Wang, Ying Liu, Jing Li, Jiajia Qi, Yonghong Zhang, Chunyan Bai, Shuang Liang

**Affiliations:** Department of Animals Sciences, College of Animal Sciences, Jilin University, Changchun, China

**Keywords:** chlorogenic acid, BPFL, porcine sertoli cells, ameliorates, impairments

## Abstract

4,4′-(9-Fluorenylidene) diphenol (BPFL, also known as BHPF and fluorene-9-bisphenol) is a novel bisphenol A substitute that is used in the plastics industry as an organic synthesis intermediate and is a potential endocrine disruptor. However, the deleterious effects of BPFL on porcine Sertoli cells (SCs) and the possible underlying mechanisms are still unclear. Chlorogenic acid (CA) is a free radical scavenger in the cellular antioxidant system that prevents oxidative damage and apoptosis. In the present research, we found that BPFL induced impairments in porcine SCs in a dose-dependent manner and that CA protected porcine SCs against BPFL exposure-induced impairments. Cell viability, proliferation and apoptosis assay results revealed that BPFL exposure could inhibit porcine SC proliferation and induce apoptosis, while CA supplementation ameliorated the effects of BPFL. Further analysis revealed that BPFL exposure induced oxidative stress, mitochondrial membrane potential dysfunction and DNA damage accumulation. Transcriptome analysis and further real-time quantitative PCR and Western blot results showed that BPFL exposure induced endoplasmic reticulum stress and apoptosis. Supplementation with CA dramatically ameliorated these phenotypes in BPFL-exposed porcine SCs. Overall, the present research reveals the possible underlying mechanisms by which BPFL exposure induced impairments and CA supplementation protected against these impairments in porcine SCs.

## Introduction

Sertoli cells (SCs), which are sustentacular cells in the mammalian testicular seminiferous tubule, play critical roles in the maintenance and regulation of spermatogenesis and provide a favorable microenvironment for sperm development (Griswold, 1998). Germ cells at different stages in the process from spermatogonial cells to sperm cells, such as primary spermatocytes and secondary spermatocytes, are embedded in the surface of the SCs and receive physical support from the SCs (França et al., 2016; Griswold, 2018). Some of the nutritional factors needed for germ cell development, such as androgen binding protein (ABP) and inhibin (INH), are synthesized and secreted by SCs (Hai et al., 2014). SCs are also involved in the formation of the blood–testis barrier (BTB), which can prevent some substances from entering the spermatogenic epithelium to form and maintain a microenvironment conducive to spermatogenesis and prevent spermatogenic antigens from escaping into the spermatogenic tubules and causing autoimmune reactions (Smith and Walker, 2014; O’Hara and Smith, 2015). Studies in different models of SC damage, such as male Fischer rats and mice, suggest that spermatogenesis is vulnerable to disruption when SCs are damaged and that targeting key SC functions can lead to rapid and massive germ cell death (Richburg and Boekelheide, 1996; Hasegawa et al., 1997). In addition, each SC can support only a limited number of germ cells; that is, the number of SCs largely determines the number of spermatozoa produced (Sharpe et al., 2003; Crisóstomo et al., 2018). Thus, SCs cultured *in vitro* are often used as a cell model for studying the testicular toxicity of drugs and compounds (Alves et al., 2014; Zhao et al., 2021).

Endocrine disruptor compounds (EDCs) are exogenous ligands that are capable of binding to cellular receptors or serum transport proteins and inducing estrogenic and/or anti-androgenic responses, interfering with the endocrine system (Liu et al., 2013; Skah et al., 2017). Bisphenols are common EDCs that are frequently encountered in daily life, including in food and water containers, plastics, feedbags, and beverage cans (Dvorakova et al., 2018). Bisphenols such as bisphenol A, E, and S have been reported in recent years to have adverse effects on male animals’ reproductive development and function because of their ubiquitous nature (Shi et al., 2019). BPFL (C_25_H_18_O_2_), also known as BHPF, is a kind of bisphenol used in the plastics industry that has estrogen-like activity and antiandrogen effects, similar to BPA and BPE (den Braver-Sewradj et al., 2020). At present, research on the reproductive toxicity of BPFL is mainly focused on oocytes or embryos from pigs, mice, and zebrafish (Jia et al., 2019; Jiao et al., 2019; Jiao et al., 2020). In addition, a recent study of humans who spend considerable time in dense industrial areas showed that their mean serum concentration of BPFL was 0.578 ng/ml and that serum BPFL was significantly correlated with the levels of the oxidative stress indices MDA and 8-OHdG. Another important finding was that the concentrations of BPFL were higher in males than in females (Gao et al., 2021), which suggests that the effect of BPFL needs to be given more attention. Although the adverse effects of BPFL on the reproductive system of female mammals and the underlying mechanism are well documented, its relationship with the reproductive system of male mammals has not yet been extensively examined.

Chlorogenic acid (CA) is a quinic acid conjugate of caffeic acid and is mainly extracted from plants such as honeysuckle, strawberry, and coffee (Gonthier et al., 2006; Naveed et al., 2018). This phytochemical constituent has a variety of biological functions, such as antioxidant activity, as well as reactive oxygen species (ROS) and free radical scavenging capacity (Priftis et al., 2018). CA was shown to regulate redox balance and inhibit mitochondrial damage by modulating Sirt1 in paraquat-treated lung epithelial cells (Kong et al., 2019). A recent study found that CA limits apoptosis by reducing ROS production and increasing intracellular glutathione levels in HepG2 cells under oxidative stress (Granado-Serrano et al., 2007). Namula et al. (2018) suggested that CA supplementation improved certain sperm parameters, including viability, plasma membrane integrity, and motility, during boar sperm freezing. Nguyen et al. (2019) found that CA supplementation during *in vitro* maturation could protect porcine oocytes from DNA damage induced by oxidative stress and improve their subsequent developmental competence after fertilization.

Herein, we hypothesized that BPFL exposure would have detrimental effects on porcine SCs and that CA supplementation would ameliorate the detrimental effects of BPFL. We adopted a porcine SC line as the model in which to explore these effects. Our results suggested that BPFL has adverse effects in porcine SCs, leading to oxidative stress, mitochondria-ER dysfunction, DNA damage and apoptosis, and that CA supplementation has potential protective effects against the impairments induced by BPFL exposure.

## Materials and Methods

### Chemicals and Reagents

BPFL (399981, purity >97%) and chlorogenic acid (C3878, purity ≥97%) were purchased from Sigma-Aldrich Chemical Company (United States). BPFL was dissolved in an appropriate amount of dimethyl sulfoxide (DMSO, 67–68–5, purity ≥99.5%), and the final concentration of DMSO was equal to that of the control treatment (0.1%). Then, cell culture medium was used to dilute BPFL to the desired concentrations before cell treatment. CA was dissolved in fetal bovine serum (FBS)-free cell culture medium. The main antibodies used were as follows: anti-CHOP rabbit pAb (1:1,000, WL00880, Wanleibio), anti-GRP78/BiP rabbit pAb (1:2,000, WL03157, Wanleibio), anti-PERK rabbit pAb (1:1,500, WL03378, Wanleibio), anti-caspase3/cleaved-caspase3 rabbit pAb (1:500, WL02117, Wanleibio), LC3A/B (D3U4C) rabbit mAb (1:1,000, 12741, Cell Signaling Technology), *β*-tubulin polyclonal antibody conjugate (1:5,000, 10094-1-AP, Proteintech), and goat anti-rabbit IgG (H + L) HRP conjugate (1:20,000, SA00001-2, Proteintech).

### Cell Culture and Cell Viability and Medication

The swine testicular (ST) cell line (ATCC^®^ CRL-1746), which has been reported as a collection of immature SCs, was purchased from ATCC (Ma et al., 2016). SCs were cultured at 37°C in a 5% CO_2_ atmosphere using DMEM (BISH1711, Biological Industries) with 10% fetal bovine serum (S711-001S, Lonsera). For the cell viability assay, when cells reached ∼60% confluence, the cell medium was replaced with fresh medium, and SCs were exposed to BPFL at different concentrations (25, 50, 75, or 100 μM), with or without CA (50, 100, 150, 200 μM). Then, 100 μl fresh medium and 10 μl CCK-8 detecting solution (CK04, Solarbio) were added to each well of a 96-well plate and incubated for 1 h at 37°C. Then, the optical density was measured by a microplate reader (Bio-Rad, United States) with absorbance at 450 nm. For further experiments, including cell proliferation assays, flow cytometry, real-time quantitative polymerase chain reaction (RT-qPCR), Western blot analyses and so on, SCs were plated at a concentration of ∼10^6^/well into six-well cell culture plates (3516, Corning). When SCs reached 70-80% confluence, the medium was replaced with fresh drug-containing medium, and the appropriate assay was performed.

### Cell Proliferation Assay

The proliferation of porcine SCs was assayed by a BeyoClick™ EdU-555 cell proliferation assay kit (C0075S, Beyotime). Briefly, the original cell medium was used to dilute 1 μl EDU to prepare EDU working fluid, the remaining cell medium was discarded, and 1 ml of fresh medium was added again. Then, the newly prepared EDU working solution was added, and the cells were incubated for 2 h at 37°C in the dark. After that, the SCs were fixed with 4% paraformaldehyde for 15 min, and after washing three times, the SCs were permeabilized with 0.3% Triton X-100 for 15 min. Then, the SCs were washed twice and incubated with 0.5 ml click additive solution in the dark for 30 min. Finally, the SCs were counterstained with 1 × Hoechst 33342 (10 μM) in the dark for 10 min, washed three times and imaged by fluorescence microscopy (TS2-S-SM, Nikon). The images were analyzed by NIH ImageJ software (National Institutes of Health, Bethesda, MD, Unites States).

### Flow Cytometry for Cell Apoptosis Detection

Porcine SC apoptosis was detected by flow cytometry using a FITC Annexin V Apoptosis Detection Kit (556547, BD Biosciences). After treatment, the cells were washed with PBS (SH30256.01B, HyClone), harvested using TrypLE Express (Gibco, Invitrogen) and centrifuged at 800 g for 6 min. Then, the cells were resuspended in 1 × binding buffer at a concentration of 1 × 10^6^ cells/ml, and 100 µL of the suspension (1 × 10^5^ cells) was transferred to a culture tube. The solution was stained with 5 µL of FITC Annexin V and 5 µL PI for 15 min at RT in the dark. After incubation, 400 µL of 1 × binding buffer was added to each tube and gently mixed with the cell suspension. The cells were analyzed by flow cytometry (FACSCalibur, BD Biosciences) within 1 h.

### Transcriptome Sequencing (RNA-Seq)

RNA-seq, which captures all mRNAs transcribed in a specific tissue or cell at a certain period, was performed with the Illumina sequencing platform. After SCs were exposed to 0.1% DMSO (control group) or 50 μM BPFL (BPFL group) for 48 h, total RNA was extracted from the control group and BPFL group. RNA quality, including integrity and total volume, was measured using an Agilent 2100 Bioanalyzer. Library preparation for transcriptome sequencing used the NEBNext^®^ Ultra™ RNA Library Prep Kit for Illumina^®^. After library quality inspection, Illumina sequencing was conducted by pooling different libraries according to the effective concentration and the demands of the target offline data volume. The basic principle of the sequencing method is sequencing-by-synthesis. Clustering of the index-coded samples was performed on a cBot Cluster Generation System using TruSeq PE Cluster Kit v3-cBot-HS (Illumina) according to the manufacturer’s instructions. After cluster generation, the library preparations were sequenced on an Illumina NovaSeq platform, and 150 bp paired-end reads were generated. After that, the Gene Ontology (GO) and Kyoto Encyclopedia of Genes and Genomes (KEGG) pathway terms assigned to the reads were compared with those of the whole transcriptome, which constituted the functional enrichment analysis.

### Detection of Intracellular Reactive Oxygen Species Levels

The generation of intracellular ROS was detected by a Reactive Oxygen Species Assay Kit (WLA070a, Wanleibio), a DCFH-DA probe. ROS in the cell can oxidize nonfluorescent DCFH to produce the strong green fluorescent substance DCF, and the resulting fluorescence intensity is proportional to the level of intracellular ROS. Briefly, 1 ml PBS was used to dilute 1 μl DCFH-DA to prepare the EdU working solution. The cells in the six-well plate were treated with the indicated compounds for 48 h. For the observation of intracellular ROS by fluorescence microscopy, the cells were washed twice, DCFH-DA working solution was added, and then the cells were incubated at 37°C for 30 min, washed with PBS twice and observed. For the detection of the fluorescence intensity of ROS, cells were harvested using TrypLE Express (12604, Gibco), and then the cells were centrifuged at 1,000 g for 6 min. After that, the cells were resuspended and diluted to a suitable density using DCFH-DA working solution, transferred to an opaque 96-well plate and incubated for 30 min. Fluorescence intensity was detected by using a SpectraMax Paradigm (SpectraMax M5 Microplate Reader, Molecular Devices), and the results were expressed as fluorescence intensity/mg protein. The whole operation was performed in the dark.

### Detection of Total Superoxide Dismutase Activity

After incubation with BPFL (50 μM) or BPFL with CA (150 μM) for 48 h, porcine SCs were harvested and washed with precooled PBS, homogenized with precooled PBS and centrifuged to obtain supernatant. Then, the enzyme activity of SOD was detected by the Total Superoxide dismutase Assay Kit with WST-8 according to the manufacturer’s instructions (S0101S, Beyotime). The absorbance was assessed at 450 nm using a microplate reader.

### Detection of DNA Damage by Comet Assay

DNA damage in individual cells was measured by the comet assay or single-cell gel electrophoresis assay (STA-350, Cell Biolabs). Briefly, porcine SCs were digested by trypsin, washed twice with ice-cold PBS, and then resuspended at 1 × 10^5^ cells/ml in ice-cold PBS. Then, comet agarose was pipetted onto the Oxiselect^TM^ comet slide to form a base layer; cells were mixed with Oxiselect comet agarose at 37°C, and then the mixture was pipetted on top of the base layer. The slide was soaked in prechilled lysis buffer for 1 h at 4°C in the dark, and after lysis, the slides were treated with alkaline electrophoresis solution for 30 min and electrophoresed for 15 min under alkaline conditions at 4°C. Porcine SCs were stained with propidium iodide (ST511, Beyotime) for 15 min in the dark, and slides were viewed by epifluorescence microscopy (TS2-S-SM, Nikon). DNA damage was quantified by measuring the displacement between the genetic material of the nucleus (“comet head”) and the resulting “tail”. Tail moment was the parameter selected to analyze the comet assay results in this study and was measured by Open Comet in ImageJ software.

### Mitochondrial Membrane Potential Assay

The cellular Mitochondrial Membrane Potential (MMP) was measured using the MMP assay kit with JC-1 (C2006, Beyotime). The proportion of mitochondrial depolarization is often measured by the relative ratio of red (JC-1 J-aggregates) to green (JC-1 monomer) fluorescence. Briefly, cells were incubated in JC-1 working solution at 37°C for 20 min. After incubation, the cells were washed three times with PBS. The red and green fluorescence signals were captured using a fluorescence microscope. The image fluorescence intensity was analyzed using NIH ImageJ software.

### RNA Extraction, Reverse Transcription and RT-qPCR

Total RNA was extracted from porcine SCs using the RNAprep Pure Cell/Bacteria Kit (DP430, TIANGEN) according to the manufacturer’s instructions. The quality and concentration of the RNA were detected with a spectrophotometer (NANODROP 2000; Thermo Scientific). Next, cDNA was synthesized by reverse transcription using the PrimeScript^TM^ RT Reagent Kit with gDNA Eraser (RR047A, Takara) according to the manufacturer’s instructions. RT-qPCR was performed on an Eppendorf AG-5341 instrument (Eppendorf, Germany) using SYBR Green Master Mix (04913914001, Roche). The primers used for RT-qPCR were designed using Primer Premier 5.0 software. The qRT-PCR primers of each gene are shown in Table 1. The relative mRNA expression levels were determined using the 2^−△△Ct^ method and normalized to TBP.

**TABLE 1:** Primers used for RT-qPCR analysis.

Symbol	Primer	Primer Sequence (5′–3′)	Gene ID (NCBI)
TBP	F-primer	GCG​ATT​TGC​TGC​TGT​AAT​CA	110259740
	R-primer	CCC​CAC​CAT​GTT​CTG​AAT​CT	
Bax	F-primer	GCC​GAA​ATG​TTT​GCT​GAC​G	396633
	R-primer	CAGCCGATCTCAAGGAAG	
Bcl-2	F-primer	CAA​GCC​TTC​AAC​CAT​TAT​CTC​AGT	100049703
	R-primer	GGG​GGG​TAA​AGA​AAA​CAG​CAT	
Caspase 8	F-primer	CCT​GGT​ATA​TCC​AAT​CAC​TGT​GC	595105
	R-primer	CTC​AGG​GTG​AAA​GTA​GGT​TGT​GG	100518913
Caspase 9	F-primer	AAC​TTC​TGC​CAT​GAG​TCG​GG	
	R-primer	CCA​AAG​CCT​GGA​CCA​TTT​GC	

### Western Blotting Analysis

Total protein was extracted from porcine SCs using RIPA lysis buffer (AR0102, Boster) with a broad-spectrum protease inhibitor mixture (AR1182, Boster) according to the manufacturer’s instructions. Then, the protein concentration of each group was measured by a BCA Protein Assay Kit (S7705, TIANGEN) according to the manufacturer’s instructions, and 30–40 µg of total protein was separated by sodium dodecyl sulfate polyacrylamide gel electrophoresis (SDS-PAGE) and transferred to a polyvinylidene difluoride (PVDF) membrane. Blocking buffer (WLA066a, Wanleibio) was used to block the transferred membranes, and the membranes were incubated overnight with primary antibodies. After washing three times with Tris-buffered saline with Tween 20 (TBST), the PVDF membranes were incubated with secondary antibodies. The immunoblots were developed using SuperSignal™ West Pico PLUS Chemiluminescent Substrate (34580, Thermo), and the signal intensities were captured by a Tanon 5200 chemiluminescence/fluorescence image analysis system. Protein levels were quantified using ImageJ software.

### Statistical Analysis

Results are expressed as means ± S.E.M. Comparative analysis among the means was performed by one-way ANOVA. Statistical analysis was carried out by SPSS 19.0 software. Each experiment was performed in triplicate. Ns *(p* > 0.05) indicates no significant difference from the control (0.1% DMSO); *(*p* < 0.05) indicates a difference from the control; and **(*p* < 0.01) indicates a significant difference from the control.

## Results

### BPFL Induces Dose-Dependent Decline in Cell Viability

**FIGURE 1:**
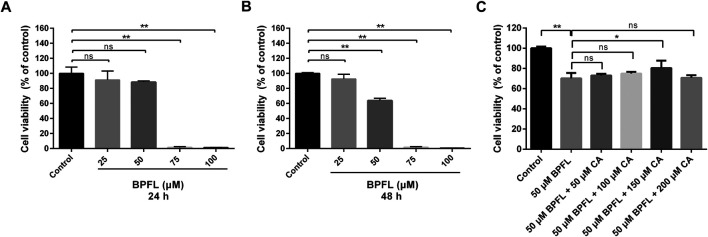
Chlorogenic acid (CA) ameliorates the BPFL exposure-induced decrease in cell viability in porcine Sertoli cells (SCs). **(A, B)** Porcine SCs were exposed to 0–100 μM for 24 and 48 h at 37°C, and cell viability was detected by CCK-8 assay respectively. **(C)** The viability of porcine SCs after 48 h of treatment with BPFL (50 μM) with/without CA (0–200 μM). (**p < 0.01) indicates a significant difference from the control (0.1% DMSO).

We first examined the effect of BPFL on SC viability. SCs were treated with BPFL at various concentrations (0–100 μM) for 24 and 48 h, as shown in Figures 1A,B. BPFL exposure reduced cell viability in a dose-dependent manner. Compared with the control group (0.1% DMSO, group: Control), the 50 μM BPFL group had nonsignificantly decreased cell viability at 24 h and significantly reduced cell viability at 48 h, whereas the viability in the 75 and 100 μM BPFL groups was dramatically decreased at both 24 and 48 h, with almost no cellular activity. Therefore, we chose 50 μM BPFL (group: BPFL) as the dose and 48 h as the treatment time. Then, CA supplementation was performed to investigate its effects on SC viability following BPFL exposure. As shown in Figure 1C, compared with the 50 μM BPFL group, 50 and 100 μM CA could restore some cell viability but not significantly, 150 μM CA significantly improved cell viability, and the effect of 200 μM CA on cell viability was not different from that of the control group. Based on these results, 50 μM BPFL and 150 μM CA (group: BPFL + CA) were used for subsequent experiments.

### Effects of Chlorogenic Acid on Cell Proliferation and Apoptosis in BPFL-Exposed Sertoli Cells

**FIGURE 2:**
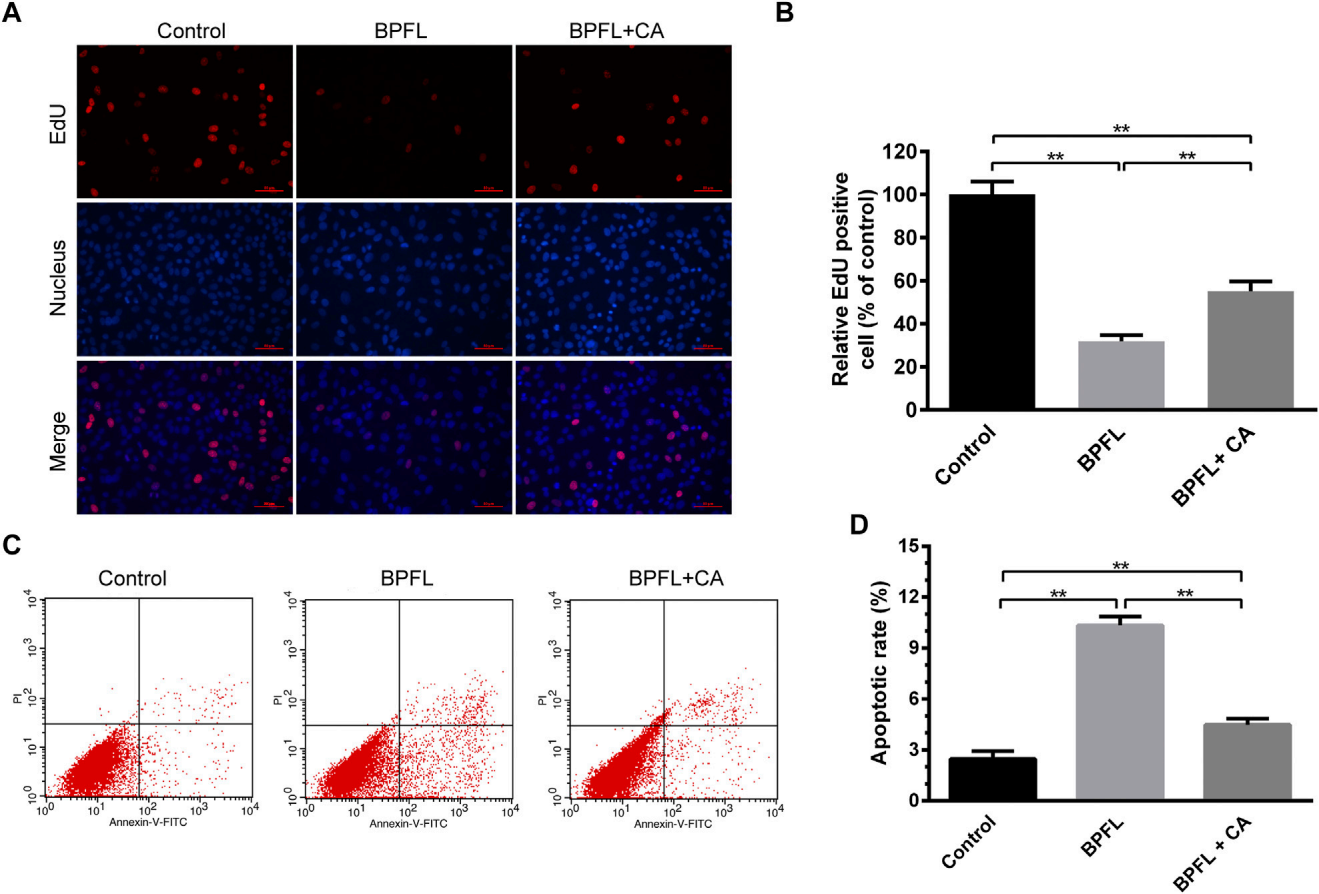
CA ameliorates BPFL exposure-induced proliferation arrest and apoptosis in porcine SCs. **(A)** Micrographs showing the incorporation of EDU in porcine SCs treated with BPFL and CA after 48 h. The proliferating cells fluoresce in red. The nuclei (Hoechst) are shown in blue. **(B)** Quantitative analysis of proliferation level was calculated by the percentage of red cells versus blue cells. **(C)** Cell apoptosis was detected by flow cytometry with Annexin V-FITC/PI double staining. **(D)** Analysis of the percentage of apoptotic cells after different treatments is shown in a histogram. (**p < 0.01) indicates a significant difference from the control (0.1% DMSO).

The thymine deoxyriboside analog EdU (5-ethynyl-2′-deoxyuridine) was incorporated in the DNA synthesis process and labeled by Alexa Fluor-555 through a click reaction to detect cell proliferation. As shown in Figures 2A,B, the proliferation of SCs was significantly decreased after 50 μM BPFL exposure, and 150 μM CA supplementation rescued the BPFL-induced decrease in the proliferation capacity of SCs. Compared with the control group, after exposure to 50 μM BPFL, the apoptosis rate of SCs was increased significantly (*p* < 0.01). In the BPFL + CA group, cells were exposed to 50 μM BPFL and 150 μM CA. As shown in Figure 2D, CA significantly attenuated BPFL-induced apoptosis. The results suggested that BPFL inhibited cell proliferation and induced cell apoptosis and that CA protected SCs from BPFL exposure.

### RNA-Seq Analysis of the Functional Effects of BPFL Exposure on Sertoli Cells

**FIGURE 3:**
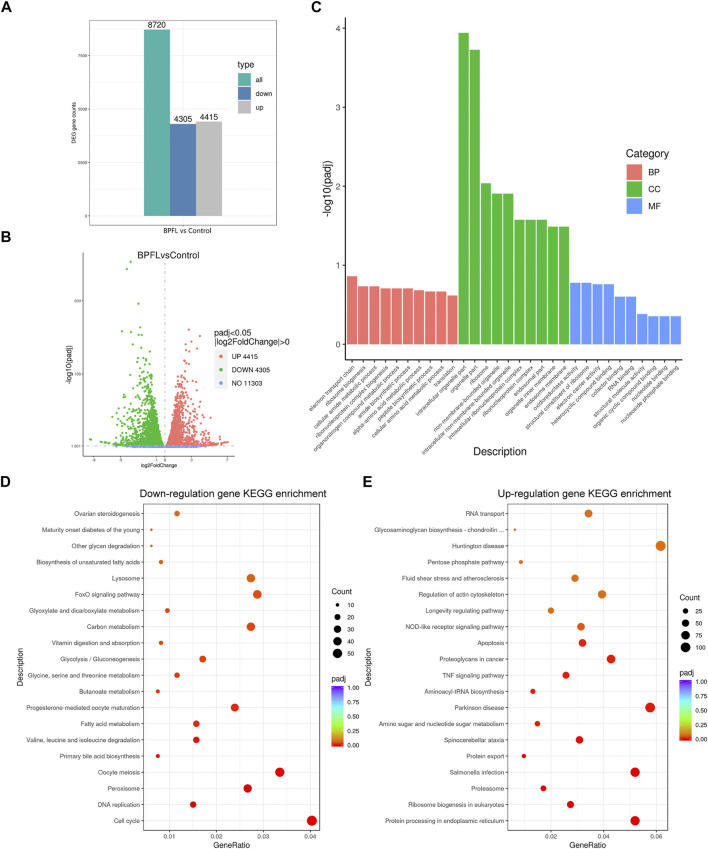
RNA-seq analysis of the effects of BPFL exposure on SCs. **(A)** The number of genes differentially expressed after BPFL treatment. **(B)** Volcano plot analysis of genes differentially expressed after BPFL treatment. **(C)** Gene Ontology enrichment analysis of the differential gene expression after BPFL treatment. **(D)** Kyoto Encyclopedia of Genes and Genomes (KEGG) enrichment analysis of genes downregulated after BPFL treatment. **(E)** KEGG enrichment analysis of genes upregulated after BPFL treatment.

To explore the functional mechanism of BPFL toxicity on SCs, the difference in general gene expression after BPFL treatment by RNA-seq was analyzed. As shown in Figure 3A, after 48 h of culture, transcriptome analysis found 8,720 genes with significantly changed expression due to BPFL treatment compared with the control group. Among these changed genes, 4,305 genes were downregulated, and 4,415 genes were upregulated (Figures 3A,B). Ten genes (five upregulated and five downregulated) that were differentially expressed after sequencing according to the PADJ value (adjusted *p*-value) were selected for fluorescence quantitative PCR to verify the accuracy of the sequencing results (Supplementary Figure S1). The number of differentially expressed genes assigned given GO terms, including biological processes, cellular components and molecular function, was analyzed. As shown in the histogram, BPFL mainly influenced the function of the electron transport chain (GO: 0022900), intracellular organelle part (GO: 0044446), and oxidoreductase activity (GO: 0016491) (Figure 3C). The results of KEGG pathway functional enrichment showed that genes downregulated in SCs upon BPFL exposure were mainly concentrated in the cell cycle (ssc04110) pathway (Figure 3D), whereas the upregulated genes were mainly enriched in protein processing in the endoplasmic reticulum (ssc04141) and peroxisome (ssc04146) and in apoptosis (ssc04210) (Figure 3E). These results provide reference information enabling us to understand the possible pathway through which BPFL induces cytotoxicity in SCs.

### Effects of Chlorogenic Acid on Oxidative Stress and DNA Damage in BPFL-Exposed Porcine Testicular SCs

**FIGURE 4:**
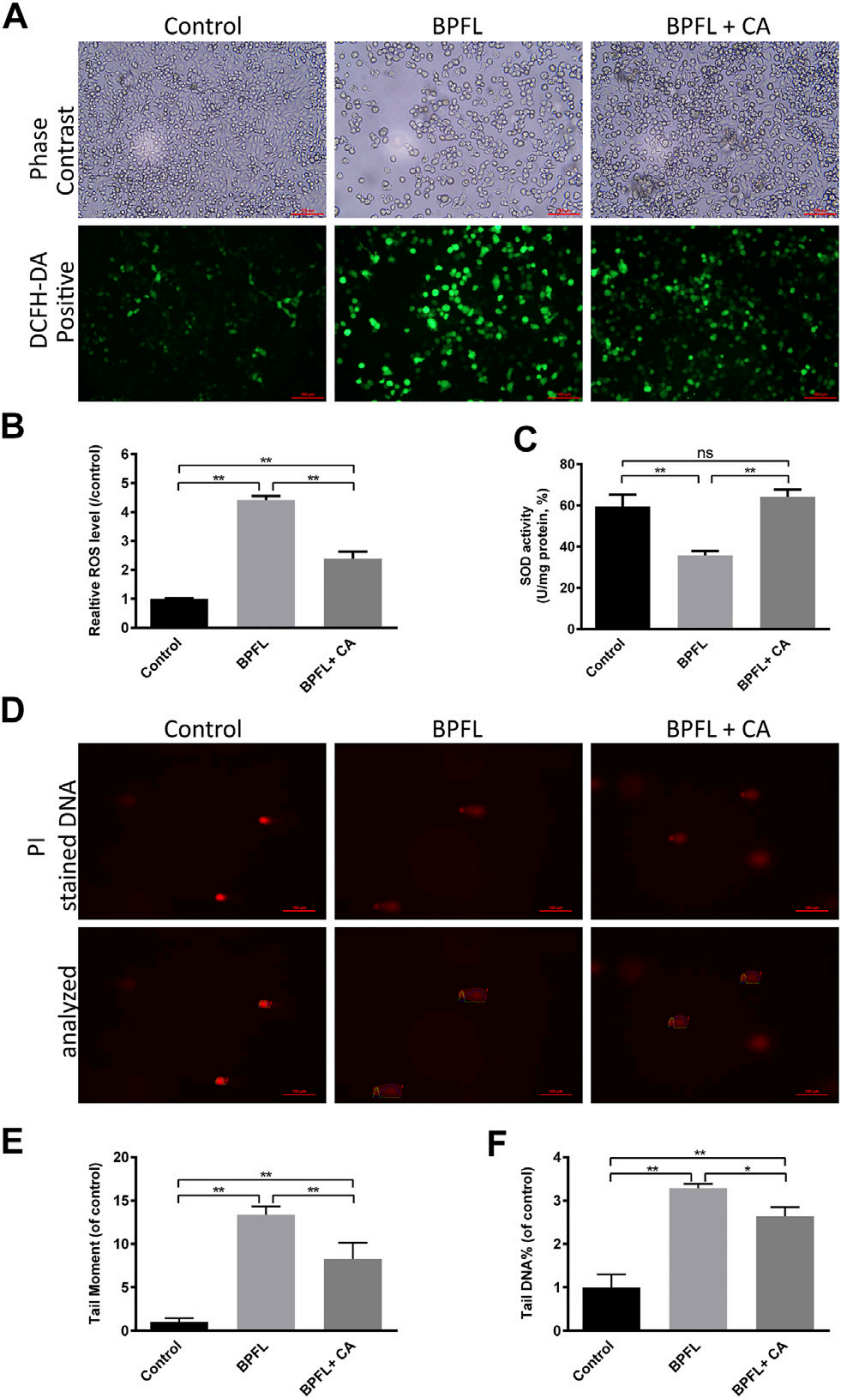
CA ameliorates BPFL exposure-induced oxidative stress and DNA damage in porcine SCs. **(A)** Fluorescence photomicrographs of reactive oxygen species in cultured SCs after BPFL and CA treatment for 48 h. **(B)** Analysis of the relative reactive oxygen species levels in different treatment groups. **(C)** SOD activity of SCs after treatment. **(D)** Representative images of DNA damage in porcine SCs after single-cell gel electrophoresis assay. **(E, F)** The tail moment values and the tail DNA% of the comet tails were analyzed by ImageJ. (*p < 0.05) indicates a difference from the control (0.1% DMSO); (**p < 0.01) indicates a significant difference from the control (0.1% DMSO).

The level of intracellular ROS was detected to reflect the degree of cellular oxidative stress. Typical fluorescent images are shown in Figure 4A. Compared with the control, treatment with BPFL (50 μM) was associated with a high fluorescence intensity, and CA rescued the ROS increase induced by BPFL. The data analysis was consistent with the results observed from the images (Figure 4B). To further determine the effects of BPFL and CA on oxidative stress, the total SOD (superoxide dismutase) activity was tested after cells were treated for 48 h. The results are shown in Figure 4C. The addition of CA ameliorated the decrease in SOD activity in the BPFL group, and SOD activity was not significantly different between the BPFL + CA group and the control group.

ROS can cause oxidative damage and attack DNA, which causes DNA damage (Meira et al., 2008). The degree of DNA damage was further evaluated by alkaline comet assay, in which damaged cellular DNA yielded a “comet tail” shape. Figure 4D shows the damaged DNA separated from intact DNA in the BPFL group. In Figures 4E,F, tail moment and tail DNA% were calculated as parameters to measure DNA damage. The two results also showed that the BPFL group had severe DNA damage and that CA supplementation significantly decreased the DNA damage levels caused by BPFL These results indicated that BPFL caused DNA damage by inducing ROS production and that CA effectively prevented this damage.

### Effects of BPFL on MMP, Endoplasmic Reticulum Stress of Sertoli Cells

**FIGURE 5:**
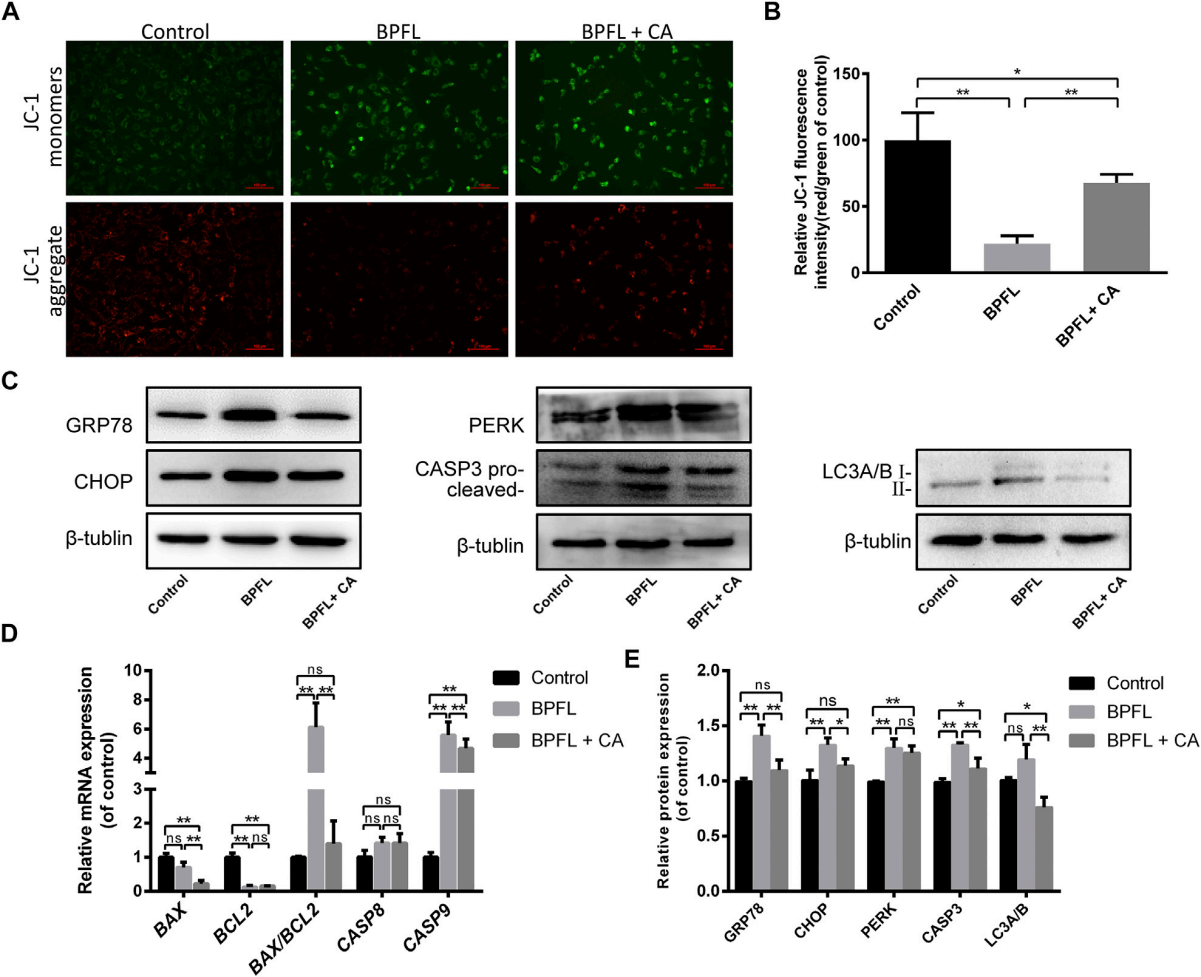
CA ameliorates BPFL exposure-induced mitochondrial dysfunction, ERS, apoptosis and autophagy in porcine SCs. **(A)** Fluorescence photomicrographs of JC-1 aggregates (green) and JC-1 monomers (red) of cultured SCs after BPFL and CA treatment for 48 h. **(B)** Analysis of the proportion of mitochondrial depolarization in different treatment groups. **(C)** Western blotting was used to detect the protein expression levels in each treatment group. **(D)** Histogram showing the relative expression of several proteins in each group compared to the reference proteins in the control group. **(E)** Relative mRNA expression of genes in different treatment groups. ns indicates no difference from the control (0.1% DMSO); (*p < 0.05) indicates a difference from the control; and (**p < 0.01) indicates a significant difference from the control.

ROS accumulation can affect cell apoptosis by depolarizing the mitochondrial membrane (Meng et al., 2021). We evaluated the change in mitochondrial membrane potential by observing the change in JC-1 fluorescence color. As shown in Figure 5A, cells in the BPFL group underwent more JC-1 fluorescence transition from red to green. Figure 5B also confirmed that the ratio of red fluorescence to green fluorescence in the BPFL group was significantly lower than that in the control group and that CA could inhibit the decrease caused by BPFL.

The results of the KEGG pathway enrichment analysis mentioned above indicated that many differentially expressed genes in the control group and the BPFL group were enriched in protein processing in endoplasmic reticulum pathways (Figure 3E). Therefore, we speculated that BPFL might cause endoplasmic reticulum stress (ERS). To test our hypothesis, the expression levels of ERS-related proteins GRP78 (78 kDa glucose regulated protein/BiP), CHOP (C/EBP-homologous protein), and PERK (protein kinase R-like ER kinase) in different treatment groups were detected by Western blot. Compared with that in the control group, the protein expression of GRP78, CHOP, and PERK was increased significantly in the BPFL group, and the protein expression of GRP78 and CHOP was not significantly changed in the BPFL + CA group. Compared with those in the BPFL group, the protein expression levels of GRP78 and CHOP were significantly decreased after CA supplementation, while PERK expression was not significantly changed. These results provide some basis for us to speculate that BPFL induces apoptosis through ERS.

Autophagy is an important mechanism for the maintenance of cell homeostasis, and the expression level of the autophagy marker LC3 (light chain 3) was detected to preliminarily explore the role of autophagy in the process of BPFL-induced apoptosis. As shown in Figures 5C,D, compared with that in the control group, LC3 expression showed an upward trend in the BPFL group, but no statistically significant difference was reached, while LC3 expression was significantly decreased in the BPFL + CA group. Compared with that in the BPFL + CA group, LC3 expression was significantly decreased in the BPFL + CA group. The results suggested that autophagy may be involved in the mitigation of BPFL-induced apoptosis by CA. In addition, we detected the expression of CASP3 (caspase 3) at the protein level and the expression of *BAX* (BCL2-associated X protein), *BCL2* (B cell lymphoma 2), *CASP8* (caspase 8), and *CASP9* (caspase 9) at the mRNA level to verify the effects of BPFL and CA on apoptosis in different respects. Compared with that in the control group, the protein expression of CASP3 (caspase 3) was increased significantly in the BPFL group and decreased in the BPFL + CA group. The ratio of the mRNA expression of the proapoptotic gene *BAX* and the antiapoptotic gene *BCL2* was increased significantly after BPFL treatment. There was no significant difference in *CASP8* expression among all groups, whereas there were significant differences in the mRNA expression of *CASP9* among all groups.

## Discussion

**FIGURE 6:**
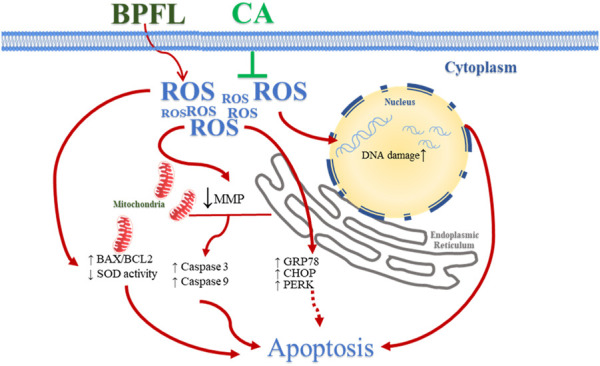
Schematic diagram illustrating the effect of CA in ameliorating BPFL exposure-induced impairments in porcine SCs. BPFL exposure impaired porcine SCs by inducing oxidative stress, mitochondrial function, DNA damage, ERS and apoptosis. CA supplementation ameliorated the impairments induced by BPFL exposure in porcine SCs.

In the present research, we used a porcine SC line as a model to investigate the protective effect of CA on porcine SCs after BPFL exposure and the potential underlying mechanism. We found that CA supplementation could alleviate the impairments of porcine SCs induced by BPFL exposure due to its antioxidant and antiapoptotic properties. The potential pathways by which CA ameliorates BPFL exposure-induced impairments in porcine SCs are summarized in Figure 6.

The effective concentration of the drug may vary in different experimental animals or cell types. Jiao et al. (2019) found that a concentration of 50 μM had no significant effect on meiosis in mouse oocytes; however, 100 or 150 μM BPFL, also known as BHPF, significantly hindered the breakdown of germinal vesicles, leading to the failure of the first polar body extrusion. Mi et al. (2019) reported that 0.1 and 10 nm BPFL had little effect on zebrafish embryonic development, whereas 1000 nM BPFL delayed the development and increased the mortality of zebrafish embryos. In the present study, the viability of porcine SCs was significantly inhibited by 50 μM BPFL, and more serious toxicity was observed at 75 and 100 μM BPFL; therefore, we chose 50 μM for subsequent experiments.

In these subsequent experiments, we found that BPFL exposure inhibits proliferation and induces apoptosis, thus impairing the *in vitro* cultured SCs. Previous studies have shown that porcine oocytes exposed to BPFL undergo early apoptosis, indicating that BPFL hinders oocyte maturation and reduces oocyte quality by inducing early apoptosis (Jia et al., 2019; Jiao et al., 2020). Our results also showed that BPFL induced apoptosis, suggesting that BPFL may have a negative effect on male fertility, raising our interest in further exploration. Then, RNA-seq was used to further explore the potential mechanisms of BPFL cytotoxicity. The RNA-seq analysis results showed that BPFL altered the expression of more than 8,000 genes in porcine SCs after BPFL exposure, reflecting that BPFL had extensive influence on transcription and translation in porcine SCs. Among the results of RNA-seq, it was shown that a large number of genes are enriched in the peroxisome (ssc04146) pathway (Figure 3D); moreover, the KEGG pathways enriched among differentially expressed genes included many metabolic pathways, such as carbon metabolism (ssc01200), amino sugar and nucleotide sugar metabolism (ssc00520) and choline metabolism in cancer (ssc05231), which showed that BPFL exposure may affect mitochondrial function (Valcarcel-Jimenez et al., 2017; van der Bliek et al., 2017). This may be because peroxisomes are essential organelles that play a crucial role in redox signaling and lipid homeostasis and many crucial metabolic processes such as free radical detoxification (Rottensteiner and Theodoulou, 2006). In the present study, by detecting the ROS content, we found that the content of ROS was increased after BPFL exposure. Our findings were consistent with those of Jia et al. (2019), who found that BPFL-exposed oocytes accumulate excess ROS and undergo oxidative stress, apoptosis, and ultimately an inability to mature.

Cellular antioxidant defenses molecules, such as the enzyme scavengers SOD (CuZnSOD, MnSOD, EC-SOD; recombinant or purified), function to eliminate excess ROS and maintain cell homeostasis (Halliwell, 2011). On this basis, we detected the activity of SOD. We found that after SC exposure to BPFL, the SOD activity was also decreased. Similar results were found in the brain tissues of male Sprague-Dawley rats exposed to quinolinic acid (1.5 mmol/kg/day) for 28 days (Loganathan and Thayumanavan, 2018) and in BJAB cells exposed to triclosan (25–75 μM) for 24 h (Alfhili et al., 2021). ROS are recognized as mediators of DNA damage (Moloney and Cotter, 2018; Srinivas et al., 2019). It has been reported that ROS can induce DNA damage directly by oxidizing nucleoside bases (formation of 8-oxoguanine), and if this damage is not repaired, G-T or G-A transversions will occur (Salehi et al., 2018). Oxidative stress can be caused by the overproduction of ROS, which can oxidize DNA and lead to DNA damage and eventually trigger cell apoptosis (Yen and Klionsky, 2008). This may be one of the pathways by which BPFL induces apoptosis in the present study.

Endogenous ROS are mainly produced by mitochondria, NADPH oxidase, peroxisomes and the endoplasmic reticulum (Meitzler et al., 2014). When cells are subjected to exogenous stressors, such as drug treatment or environmental contamination, they produce ROS through the above organelles or enzymes (Ziech et al., 2011; Meitzler et al., 2014). Mitochondria are the main sites of ROS production and the main organelles attacked by ROS (Chen et al., 2003; Grivennikova and Vinogradov, 2006). In this study, we found that BPFL induced an increase in ROS content increased and a decrease in mitochondrial membrane potential. In addition, the upregulated expression ratio of *BAX* to *BCL2* and the upregulated expression levels of *CASP3* and *CASP9* in this study are also consistent with the mitochondrial pathway of apoptosis activated by ROS described in a previous study by Redza-Dutordoir et al. (2016). Other mechanisms that may be involved in this process, such as the release of calcium homeostasis and the activation of JNK pathways (Putcha et al., 2003; Orrenius et al., 2015), are also worth further exploration in subsequent studies. According to the results of our study, we hypothesized that BPFL induces oxidative stress and mitochondrial damage, which is one of the causes of apoptosis in SCs.

In addition to the previously mentioned mitochondrial pathways, oxidative stress also induces cell surface death receptor pathways and endoplasmic reticulum pathways, leading to downstream caspase activation and ultimately to cell apoptosis (Jin and El-Deiry, 2005). In the present study, we used RNA-seq to identify the differential expression of several genes associated with protein processing in the endoplasmic reticulum (ssc04141) during BPFL-induced SC damage. The phenomenon of endoplasmic reticulum homeostasis being disrupted by adverse environmental stimuli or drug treatment and resulting in the accumulation of luminal misfolded and unfolded proteins in the ER is known as ERS (Haynes et al., 2004). The unfolded protein response (UPR) protects cells from short-term or mild ERS-induced damage (Simard et al., 2016), which is mediated by an endoplasmic reticulum chaperone (GRP78) and three ERS receptor proteins (PERK, ATF6, and IRE) (Scheuner et al., 2001). Some reports have shown that oxidative stress can cause ERS (Haynes et al., 2004). On the other hand, ERS can also cause ROS production (Malhotra and Kaufman, 2007). When severe or prolonged ERS fails to restore the stability of the internal environment in a timely manner, downstream apoptotic signaling molecules such as CHOP can be activated to clear damaged cells (Rutkowski et al., 2006). There is evidence that the accumulation of CHOP can promote apoptosis by inhibiting several antiapoptotic proteins in the BCL2 family. ERS induced by persistent oxidative stress leads to the activation of CHOP and further production of additional ROS (Zeeshan et al., 2016), which is a vicious cycle. In our study, the upregulated expression levels of PERK and GRP78 proteins confirmed that BPFL induced ERS, and the upregulation of CHOP expression indicated that BPFL could induce ERS-mediated apoptosis, which is consistent with the observation by Yang et al. (2015) that NaF induces apoptosis through ERS mediated by ROS in rat SCs.

Autophagy is another mode of cell death that is mediated by oxidative stress. Some external stimuli that cause apoptosis can also lead to autophagy (Maiuri et al., 2007). It has also been reported that autophagy is usually an adaptive pathway to promote cell survival under stress conditions or as a defense mechanism against different environmental stresses (Chiarelli et al., 2016). According to the different degradation pathways, there are three types of autophagy, namely, macroautophagy, microautophagy and chaperone-mediated autophagy (Mizushima, 2007). Although their mechanisms differ, they are all related to lysosomal degradation. “Autophagy” usually refers to macroautophagy, which is the most studied kind of autophagy at present (Mizushima, 2007). In macroautophagy, cells form autophagosomes by wrapping proteins, organelles and other components that need to be degraded in a bilayer membrane structure; these autophagosomes fuse with lysosomes to form autophagy lysosomes, in which the internal components of autophagosomes are degraded (Mizushima, 2007). Microtubule-associated protein light chain 3 (LC3) was the first identified autophagosome-labeling protein. It exists in two forms, LC3-I and LC3-II, and has been used as a specific marker for autophagy activity monitoring (Kabeya et al., 2000; Mizushima et al., 2008). In this study, as increased oxidative stress was observed in BPFL-treated SCs, we argued that BPFL may induce an autophagic mode of cell death, but no significant increase in LC3 expression was observed in the 50 μM-treated cells compared to the control group, indicating that the effect of BPFL on autophagy of SCs was weak. This was similar to the results obtained when endosulfan was added to Sertoli germ cells (Rastogi et al., 2014). In contrast to the results in this study, BPFL was found to induce increased expression of the autophagosome-forming protein LC3B in oocytes, which leads to the failure of *in vitro* maturation of oocytes (Jia et al., 2019; Jiao et al., 2019). The effect of BPFL on autophagy may be due to the cell type or other potential molecular mechanisms, which will be studied in the future.

CA is a kind of polyphenolic substance that can be extracted from many plant species (Naveed et al., 2018). CA scavenges free radicals *in vitro* and prevents the spread of the oxidation process, and it is an effective scavenger of ROS (Priftis et al., 2018). For instance, the addition of CA to rat pheochromocytoma cells protected the cells from H_2_O_2_-mediated oxidative damage (Yao et al., 2019). The results of this study showed that CA can reduce BPFL-induced ROS production in SCs and mitigate BPFL-induced apoptosis through ROS-mediated mitochondrial and endoplasmic reticulum pathways. In this process, the antioxidant effect and cell protection effect of CA in SCs were similar to those of paraquat in A549 cells (Kong et al., 2019), dexamethasone in osteoblastic cells (Han et al., 2019), and 6-hydroxydopamine in SH-SY5Y cells (Shan et al., 2019). In this experiment, the function of CA was consistent with that of CA in improving Cd-induced kidney and liver injury, such as, increasing SOD and CAT activities and improving the balance of redox reactions (Ding et al., 2021).

## Conclusion

In conclusion, these results suggest that BPFL exposure induces ROS production, causes mitochondrial function damage and ERS, and causes caspase activation, which eventually leads to cell apoptosis. In addition, because of its antioxidant properties, CA could reduce the accumulation of ROS induced by BPFL and ultimately reduce the impairments of SC exposure to BPFL. In the future, animal experiments should be conducted to confirm the effect of CA supplementation on ameliorating BPFL exposure-induced impairments in porcine testicular SCs and reduce the limitations of cell tests.

## Data Availability

The RNA sequencing data has been deposited into BioProject, NCBI (Accession: PRJNA713955).

## References

[B1] Alfhili M. A. Hussein H. A. M. Park Y. Lee M. H. Akula S. M. (2021). Triclosan Induces Apoptosis in Burkitt Lymphoma-Derived BJAB Cells through Caspase and JNK/MAPK Pathways. Apoptosis 26, 96–110. 10.1007/s10495-020-01650-0 33387145

[B2] Alves M. G. Martins A. D. Vaz C. V. Correia S. Moreira P. I. Oliveira P. F. (2014). Metformin and Male Reproduction: Effects on Sertoli Cell Metabolism. Br. J. Pharmacol. 171 (4), 1033–1042. 10.1111/bph.12522 24261663 PMC3925041

[B3] Chen Q. Vazquez E. J. Moghaddas S. Hoppel C. L. Lesnefsky E. J. (2003). Production of Reactive Oxygen Species by Mitochondria. J. Biol. Chem. 278 (38), 36027–36031. 10.1074/jbc.M304854200 12840017

[B4] Chiarelli R. Martino C. Agnello M. Bosco L. Roccheri M. C. (2016). Autophagy as a Defense Strategy Against Stress: Focus on *Paracentrotus lividus* Sea Urchin Embryos Exposed to Cadmium. Cell Stress and Chaperones 21 (1), 19–27. 10.1007/s12192-015-0639-3 26362931 PMC4679740

[B5] Crisóstomo L. Alves M. G. Gorga A. Sousa M. Riera M. F. Galardo M. N. (2018). Molecular Mechanisms and Signaling Pathways Involved in the Nutritional Support of Spermatogenesis by Sertoli Cells. Methods Mol. Biol. 1748, 129–155. 10.1007/978-1-4939-7698-0_11 29453570

[B6] den Braver-Sewradj S. P. van Spronsen R. Hessel E. V. S. (2020). Substitution of Bisphenol A: a Review of the Carcinogenicity, Reproductive Toxicity, and Endocrine Disruption Potential of Alternative Substances. Crit. Rev. Toxicol. 50 (2), 128–147. 10.1080/10408444.2019.1701986 32031044

[B7] Ding Y. Li X. Liu Y. Wang S. Cheng D. (2021). Protection Mechanisms Underlying Oral Administration of Chlorogenic Acid Against Cadmium-Induced Hepatorenal Injury Related to Regulating Intestinal Flora Balance. J. Agric. Food Chem. 69 (5), 1675–1683. 10.1021/acs.jafc.0c06698 33494608

[B8] Dvorakova M. Kejlová K. Rucki M. Jírová D. (2018). Selected Bisphenols and Phthalates Screened for Estrogen and Androgen Disruption by In Silico and *In Vitro* Methods. Neuro Endocrinol. Lett. 39 (5), 409–416. 10.1371/journal.pone.0100952 30664347

[B9] França L. R. Hess R. A. Dufour J. M. Hofmann M. C. Griswold M. D. (2016). The Sertoli Cell: One Hundred Fifty Years of Beauty and Plasticity. Andrology 4 (2), 189–212. 10.1111/andr.12165 26846984 PMC5461925

[B10] Gao C. He H. Qiu W. Zheng Y. Chen Y. Hu S. (2021). Oxidative Stress, Endocrine Disturbance, and Immune Interference in Humans Showed Relationships to Serum Bisphenol Concentrations in a Dense Industrial Area. Environ. Sci. Technol. 55 (3), 1953–1963. 10.1021/acs.est.0c07587 33496180

[B11] Gonthier M.-P. Remesy C. Scalbert A. Cheynier V. Souquet J.-M. Poutanen K. (2006). Microbial Metabolism of Caffeic Acid and its Esters Chlorogenic and Caftaric Acids by Human Faecal Microbiota *In Vitro* . Biomed. Pharmacother. 60 (9), 536–540. 10.1016/j.biopha.2006.07.084 16978827

[B12] Granado-Serrano A. B. Angeles Martín M. Izquierdo-Pulido M. Goya L. Bravo L. Ramos S. (2007). Molecular Mechanisms of (−)-Epicatechin and Chlorogenic Acid on the Regulation of the Apoptotic and Survival/Proliferation Pathways in a Human Hepatoma Cell Line. J. Agric. Food Chem. 55 (5), 2020–2027. 10.1021/jf062556x 17286412

[B13] Griswold M. D. (2018). 50 Years of Spermatogenesis: Sertoli Cells and Their Interactions With Germ Cells. Biol. Reprod. 99 (1), 87–100. 10.1093/biolre/ioy027 29462262 PMC7328471

[B14] Griswold M. D. (1998). The Central Role of Sertoli Cells in Spermatogenesis. Semin. Cel Develop. Biol. 9 (4), 411–416. 10.1006/scdb.1998.0203 9813187

[B15] Grivennikova V. G. Vinogradov A. D. (2006). Generation of Superoxide by the Mitochondrial Complex I. Biochim. Biophys. Acta (Bba) - Bioenerg. 1757 (5–6), 553–561. 10.1016/j.bbabio.2006.03.013 16678117

[B16] Hai Y. Hou J. Liu Y. Liu Y. Yang H. Li Z. (2014). The Roles and Regulation of Sertoli Cells in Fate Determinations of Spermatogonial Stem Cells and Spermatogenesis. Semin. Cel Develop. Biol. 29, 66–75. 10.1016/j.semcdb.2014.04.007 24718316

[B17] Halliwell B. (2011). Free Radicals and Antioxidants - Quo Vadis?. Trends Pharmacol. Sci. 32 (3), 125–130. 10.1016/j.tips.2010.12.002 21216018

[B18] Han D. Gu X. Gao J. Wang Z. Liu G. Barkema H. W. (2019). Chlorogenic Acid Promotes the Nrf2/HO-1 Anti-oxidative Pathway by Activating p21Waf1/Cip1 to Resist Dexamethasone-Induced Apoptosis in Osteoblastic Cells. Free Radic. Biol. Med. 137, 1–12. 10.1016/j.freeradbiomed.2019.04.014 31004750

[B19] Hasegawa M. Wilson G. Russell L. D. Meistrich M. L. (1997). Radiation-induced Cell Death in the Mouse Testis: Relationship to Apoptosis. Radiat. Res. 147 (4), 457–467. 10.2307/3579503 9092926

[B20] Haynes C. M. Titus E. A. Cooper A. A. (2004). Degradation of Misfolded Proteins Prevents ER-Derived Oxidative Stress and Cell Death. Mol. Cel 15 (5), 767–776. 10.1016/j.molcel.2004.08.025 15350220

[B21] Jia Z. Wang H. Feng Z. Zhang S. Wang L. Zhang J. (2019). Fluorene-9-bisphenol Exposure Induces Cytotoxicity in Mouse Oocytes and Causes Ovarian Damage. Ecotoxicology Environ. Saf. 180, 168–178. 10.1016/j.ecoenv.2019.05.019 31082581

[B22] Jiao X.-F. Liang Q.-M. Wu D. Ding Z.-M. Zhang J.-Y. Chen F. (2019). Effects of Acute Fluorene-9-Bisphenol Exposure on Mouse Oocyte *In Vitro* Maturation and its Possible Mechanisms. Environ. Mol. Mutagen. 60 (3), 243–253. 10.1002/em.22258 30499614

[B23] Jiao X. Ding Z. Meng F. Zhang X. Wang Y. Chen F. (2020). The Toxic Effects of Fluorene‐9‐bisphenol on Porcine Oocyte *In Vitro* Maturation. Environ. Toxicol. 35 (2), 152–158. 10.1002/tox.22851 31696613

[B24] Jin Z. El-Deiry W. S. (2005). Overview of Cell Death Signaling Pathways. Cancer Biol. Ther. 4 (2), 147–171. 10.4161/cbt.4.2.1508 15725726

[B25] Kabeya Y. Mizushima N. Ueno T. Yamamoto A. Kirisako T. Noda T. (2000). LC3, a Mammalian Homologue of Yeast Apg8p, Is Localized in Autophagosome Membranes After Processing. EMBO J. 19 (21), 5720–5728. 10.1093/emboj/19.21.5720 11060023 PMC305793

[B26] Kong D. Ding Y. Liu J. Liu R. Zhang J. Zhou Q. (2019). Chlorogenic Acid Prevents Paraquat-Induced Apoptosis *via* Sirt1-Mediated Regulation of Redox and Mitochondrial Function. Free Radic. Res. 53 (6), 680–693. 10.1080/10715762.2019.1621308 31106605

[B27] Liu C. Duan W. Li R. Xu S. Zhang L. Chen C. (2013). Exposure to Bisphenol A Disrupts Meiotic Progression During Spermatogenesis in Adult Rats through Estrogen-Like Activity. Cell Death Dis 4, e676. 10.1038/cddis.2013.203 23788033 PMC3702305

[B28] Loganathan C. Thayumanavan P. (2018). Asiatic Acid Prevents the Quinolinic Acid-Induced Oxidative Stress and Cognitive Impairment. Metab. Brain Dis. 33 (1), 151–159. 10.1007/s11011-017-0143-9 29086235

[B29] Ma C. Song H. Guan K. Zhou J. Xia X. Li F. (2016). Characterization of Swine Testicular Cell Line as Immature Porcine Sertoli Cell Line. In Vitro Cell.Dev.Biol.-Animal 52 (4), 427–433. 10.1007/s11626-015-9994-8 26744029

[B30] Maiuri M. C. Zalckvar E. Kimchi A. Kroemer G. (2007). Self-Eating and Self-Killing: Crosstalk Between Autophagy and Apoptosis. Nat. Rev. Mol. Cel Biol 8 (9), 741–752. 10.1038/nrm2239 17717517

[B31] Malhotra J. D. Kaufman R. J. (2007). The Endoplasmic Reticulum and the Unfolded Protein Response. Semin. Cel Develop. Biol. 18 (6), 716–731. 10.1016/j.semcdb.2007.09.003 PMC2706143 18023214

[B32] Meira L. B. Bugni J. M. Green S. L. Lee C.-W. Pang B. Borenshtein D. (2008). DNA Damage Induced by Chronic Inflammation Contributes to Colon Carcinogenesis in Mice. J. Clin. Invest. 118 (7), 2516–2525. 10.1172/JCI35073 18521188 PMC2423313

[B33] Meitzler J. L. Antony S. Wu Y. Juhasz A. Liu H. Jiang G. (2014). NADPH Oxidases: a Perspective on Reactive Oxygen Species Production in Tumor Biology. Antioxid. Redox Signaling 20 (17), 2873–2889. 10.1089/ars.2013.5603 PMC4026372 24156355

[B34] Meng L. Liu J. Wang C. Ouyang Z. Kuang J. Pang Q. (2021). Sex-Specific Oxidative Damage Effects Induced by BPA and its Analogs on Primary Hippocampal Neurons Attenuated by EGCG. Chemosphere 264 (Pt 1), 128450. 10.1016/j.chemosphere.2020.128450 33007573

[B35] Mi P. Zhang Q.-P. Li S.-B. Liu X.-Y. Zhang S.-H. Li M. (2019). Melatonin Protects Embryonic Development and Maintains Sleep/Wake Behaviors From the Deleterious Effects of Fluorene-9-Bisphenol in Zebrafish (*Danio rerio*). J. Pineal Res. 66 (1), e12530. 10.1111/jpi.12530 30269372

[B36] Mizushima N. (2007). Autophagy: Process and Function. Genes Dev. 21 (22), 2861–2873. 10.1101/gad.1599207 18006683

[B37] Mizushima N. Levine B. Cuervo A. M. Klionsky D. J. (2008). Autophagy Fights Disease Through Cellular Self-Digestion. Nature 451 (7182), 1069–1075. 10.1038/nature06639 18305538 PMC2670399

[B38] Moloney J. N. Cotter T. G. (2018). ROS Signalling in the Biology of Cancer. Semin. Cel Develop. Biol. 80, 50–64. 10.1016/j.semcdb.2017.05.023 28587975

[B39] Namula Z. Hirata M. Wittayarat M. Tanihara F. Thi Nguyen N. Hirano T. (2018). Effects of Chlorogenic Acid and Caffeic Acid on the Quality of Frozen‐Thawed Boar Sperm. Reprod. Dom Anim. 53 (6), 1600–1604. 10.1111/rda.13288 30053311

[B40] Naveed M. Hejazi V. Abbas M. Kamboh A. A. Khan G. J. Shumzaid M. (2018). Chlorogenic Acid (CGA): A Pharmacological Review and Call for Further Research. Biomed. Pharmacother. 97, 67–74. 10.1016/j.biopha.2017.10.064 29080460

[B41] Nguyen T. V. Do L. T. K. Somfai T. Otoi T. Taniguchi M. Kikuchi K. (2019). Presence of Chlorogenic Acid During *In Vitro* Maturation Protects Porcine Oocytes From the Negative Effects of Heat Stress. Anim. Sci. J. 90 (12), 1530–1536. 10.1111/asj.13302 31663235

[B42] O’Hara L. Smith L. B. (2015). Androgen Receptor Roles in Spermatogenesis and Infertility. Best Pract. Res. Clin. Endocrinol. Metab. 29 (4), 595–605. 10.1016/j.beem.2015.04.006 26303086

[B43] Orrenius S. Gogvadze V. Zhivotovsky B. (2015). Calcium and Mitochondria in the Regulation of Cell Death. Biochem. Biophysical Res. Commun. 460 (1), 72–81. 10.1016/j.bbrc.2015.01.137 25998735

[B44] Priftis A. Panagiotou E.-M. Lakis K. Plika C. Halabalaki M. Ntasi G. (2018). Roasted and green Coffee Extracts Show Antioxidant and Cytotoxic Activity in Myoblast and Endothelial Cell Lines in a Cell Specific Manner. Food Chem. Toxicol. 114, 119–127. 10.1016/j.fct.2018.02.029 29452189

[B45] Putcha G. V. Le S. Frank S. Besirli C. G. Clark K. Chu B. (2003). JNK-Mediated BIM Phosphorylation Potentiates BAX-Dependent Apoptosis. Neuron 38 (6), 899–914. 10.1016/s0896-6273(03)00355-6 12818176

[B46] Rastogi D. Narayan R. Saxena D. K. Chowdhuri D. K. (2014). Endosulfan Induced Cell Death in Sertoli-Germ Cells of Male Wistar Rat Follows Intrinsic Mode of Cell Death. Chemosphere 94, 104–115. 10.1016/j.chemosphere.2013.09.029 24125708

[B47] Redza-Dutordoir M. Averill-Bates D. A. (2016). Activation of Apoptosis Signalling Pathways by Reactive Oxygen Species. Biochim. Biophys. Acta (Bba) - Mol. Cel Res. 1863 (12), 2977–2992. 10.1016/j.bbamcr.2016.09.012 27646922

[B48] Richburg J. H. Boekelheide K. (1996). Mono-(2-ethylhexyl) Phthalate Rapidly Alters Both Sertoli Cell Vimentin Filaments and Germ Cell Apoptosis in Young Rat Testes. Toxicol. Appl. Pharmacol. 137 (1), 42–50. 10.1006/taap.1996.0055 8607140

[B49] Rottensteiner H. Theodoulou F. L. (2006). The Ins and Outs of Peroxisomes: Co-ordination of Membrane Transport and Peroxisomal Metabolism. Biochim. Biophys. Acta (Bba) - Mol. Cel Res. 1763 (12), 1527–1540. 10.1016/j.bbamcr.2006.08.012 17010456

[B50] Rutkowski D. T. Arnold S. M. Miller C. N. Wu J. Li J. Gunnison K. M. (2006). Adaptation to ER Stress Is Mediated by Differential Stabilities of Pro-Survival and Pro-Apoptotic mRNAs and Proteins. Plos Biol. 4 (11), e374. 10.1371/journal.pbio.0040374 17090218 PMC1634883

[B51] Salehi F. Behboudi H. Kavoosi G. Ardestani S. K. (2018). Oxidative DNA Damage Induced by ROS-Modulating Agents With the Ability to Target DNA: A Comparison of the Biological Characteristics of Citrus Pectin and Apple Pectin. Sci. Rep. 8 (1), 13902. 10.1038/s41598-018-32308-2 30224635 PMC6141541

[B52] Scheuner D. Song B. McEwen E. Liu C. Laybutt R. Gillespie P. (2001). Translational Control Is Required for the Unfolded Protein Response and *In Vivo* Glucose Homeostasis. Mol. Cel 7 (6), 1165–1176. 10.1016/s1097-2765(01)00265-9 11430820

[B53] Shan S. Tian L. Fang R. (2019). Chlorogenic Acid Exerts Beneficial Effects in 6-Hydroxydopamine-Induced Neurotoxicity by Inhibition of Endoplasmic Reticulum Stress. Med. Sci. Monit. 25, 453–459. 10.12659/MSM.911166 30645211 PMC6342059

[B54] Sharpe R. McKinnell C. Kivlin C. Fisher J. (2003). Proliferation and Functional Maturation of Sertoli Cells, and Their Relevance to Disorders of Testis Function in Adulthood. Reproduction 125 (6), 769–784. 10.1530/rep.0.1250769 12773099

[B55] Shi M. Whorton A. E. Sekulovski N. MacLean J. A. Hayashi K. (2019). Prenatal Exposure to Bisphenol A, E, and S Induces Transgenerational Effects on Male Reproductive Functions in Mice. Toxicol. Sci. 172 (2), 303–315. 10.1093/toxsci/kfz207 31532523

[B56] Simard J.-C. Durocher I. Girard D. (2016). Silver Nanoparticles Induce Irremediable Endoplasmic Reticulum Stress Leading to Unfolded Protein Response Dependent Apoptosis in Breast Cancer Cells. Apoptosis 21 (11), 1279–1290. 10.1007/s10495-016-1285-7 27586505

[B57] Skah S. Uchuya-Castillo J. Sirakov M. Plateroti M. (2017). The Thyroid Hormone Nuclear Receptors and the Wnt/β-Catenin Pathway: An Intriguing Liaison. Develop. Biol. 422 (2), 71–82. 10.1016/j.ydbio.2017.01.003 28069375

[B58] Smith L. B. Walker W. H. (2014). The Regulation of Spermatogenesis by Androgens. Semin. Cel Develop. Biol. 30, 2–13. 10.1016/j.semcdb.2014.02.012 PMC4043871 24598768

[B59] Srinivas U. S. Tan B. W. Q. Vellayappan B. A. Jeyasekharan A. D. (2019). ROS and the DNA Damage Response in Cancer. Redox Biol. 25, 101084. 10.1016/j.redox.2018.101084 30612957 PMC6859528

[B60] Valcarcel-Jimenez L. Gaude E. Torrano V. Frezza C. Carracedo A. (2017). Mitochondrial Metabolism: Yin and Yang for Tumor Progression. Trends Endocrinol. Metab. 28 (10), 748–757. 10.1016/j.tem.2017.06.004 28938972 PMC6047739

[B61] van der Bliek A. M. Sedensky M. M. Morgan P. G. (2017). Cell Biology of the Mitochondrion. Genetics 207 (3), 843–871. 10.1534/genetics.117.300262 29097398 PMC5676242

[B62] Yang Y. Lin X. Huang H. Feng D. Ba Y. Cheng X. (2015). Sodium Fluoride Induces Apoptosis Through Reactive Oxygen Species-Mediated Endoplasmic Reticulum Stress Pathway in Sertoli Cells. J. Environ. Sci. 30, 81–89. 10.1016/j.jes.2014.11.004 25872712

[B63] Yao J. Peng S. Xu J. Fang J. (2019). Reversing ROS‐Mediated Neurotoxicity by Chlorogenic Acid Involves its Direct Antioxidant Activity and Activation of Nrf2‐ARE Signaling Pathway. Biofactors 45 (4), 616–626. 10.1002/biof.1507 30951611

[B64] Yen W.-L. Klionsky D. J. (2008). How to Live Long and Prosper: Autophagy, Mitochondria, and Aging. Physiology 23, 248–262. 10.1152/physiol.00013.2008 18927201

[B65] Zeeshan H. Lee G. Kim H.-R. Chae H.-J. (2016). Endoplasmic Reticulum Stress and Associated ROS. Int. J. Mol. Sci. 17 (3), 327. 10.3390/ijms17030327 26950115 PMC4813189

[B66] Zhao S. Yuan C. Tuo X. Zhou C. Zhao Q. Shen T. (2021). MCLR Induces Dysregulation of Calcium Homeostasis and Endoplasmic Reticulum Stress Resulting in Apoptosis in Sertoli Cells. Chemosphere 263, 127868. 10.1016/j.chemosphere.2020.127868 32828052

[B67] Ziech D. Franco R. Pappa A. Panayiotidis M. I. (2011). Reactive Oxygen Species (ROS)—induced Genetic and Epigenetic Alterations in Human Carcinogenesis. Mutat. Research/Fundamental Mol. Mech. Mutagenesis 711 (1–2), 167–173. 10.1016/j.mrfmmm.2011.02.015 21419141

