# Supplementary material for: Chlorogenic Acid Ameliorates Damage Induced by Fluorene-9-Bisphenol in Porcine Sertoli Cells
Source: Front Pharmacol. 2021 Jun 9;12:678772. doi: 10.3389/fphar.2021.678772 (PMC8219976; doi:10.3389/fphar.2021.678772)
Supplement: Supplementary file 1 [file Table1.DOCX]

**Figure Legend**

**FIGURE SUPPLEMENTAL 1** RT-qPCR was used to verify the sequencing results. The results showed that the RT-qPCR results of the 10 selected genes were basically consistent with the results of RNA-seq analysis, which proved the reliability of RNA-Seq sequencing analysis results.

| *Symbol* | *Primer* | | *Primer Sequence(5′–3′)* | *Gene ID (NCBI)* |
| --- | --- | --- | --- | --- |
| *Up-regulated* |  |  | |  |
| *CREB3L1* | F-Primer  R-Primer | CACCAGGACGAAGCACAA  TTACATCCGAGAACAACGAAC | | 100312973 |
| *GPRC5A*  *LAMB3* | F-Primer  R -Primer  F-Primer  R -Primer | ATGCCGCTGATGAGAAAG  TCACCAAGCAACGGAACA  CACATCGTTCTGCGACTGC  ACTGACCAAGCCTGAGACCTAC | | 100624186  100736655 |
| *SRXN1* | F-Primer  R-Primer | CGCTGAAGCTGCTGGTAGG  TCCACTCGGGTTGTATCGC | | 100736655 |
| *CCL5* | F-Primer  R-Primer | ACTTGCTGCTGGTGTAGAAATA  TGCCCTTGCTGTCATCCT | | 396613 |
| *Down-regulated* |  |  | |  |
| *CLDN8*  *DIO1* | F-Primer  R -Primer  F-Primer  R -Primer | CCAGCAGGGAATCGTAGA  TTGGTGGTGTTGGAATGG  TCCTGACAGGCTGACCAC  ACCCTCACTTCTCCCACG | | 100302021  414380 |
| *HNF4A*  *KDM2B*  *DAO* | F-Primer  R -Primer  F-Primer  R -Primer  F-Primer  R -Primer | TTCCTCTTGTCTTTGTCCACC  CACAGGCAAACACTACGGG  GTGGACTGGGTGGACAACA  ACCAAACGGAAGTGCCTC  GTGGACTGGGTGGACAACA  ACCAAACGGAAGTGCCTC | | 733636  100512074  397134 |

RT-qPCR Primers Used for RNA-seq
