# Supplementary figures and images for: Chlorogenic Acid Ameliorates Damage Induced by Fluorene-9-Bisphenol in Porcine Sertoli Cells
Source: Front Pharmacol. 2021 Jun 9;12:678772. doi: 10.3389/fphar.2021.678772 (PMC8219976; doi:10.3389/fphar.2021.678772)

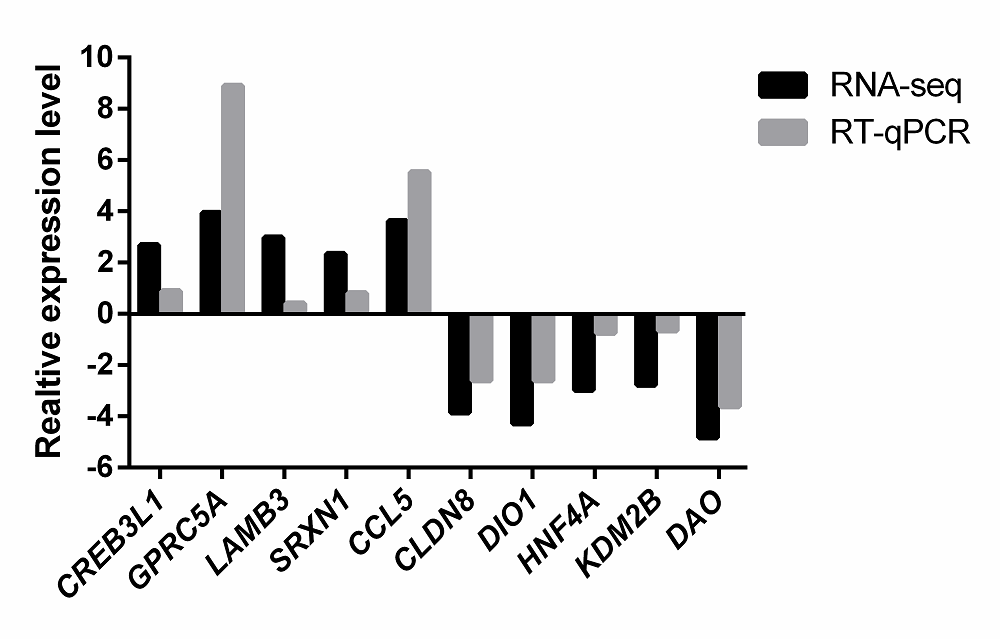

Supplement: Supplementary file 2 [file Image1.TIF]
